# Transitions of Care from Child and Adolescent Mental Health Services to Adult Mental Health Services (TRACK Study): A study of protocols in Greater London

**DOI:** 10.1186/1472-6963-8-135

**Published:** 2008-06-23

**Authors:** Swaran P Singh, Moli Paul, Tamsin Ford, Tami Kramer, Tim Weaver

**Affiliations:** 1 Warwick Medical School, University of Warwick, Coventry, CV4 7AL, UK; 2 Institute for Health Service Research, Peninsula College of Medicine and Dentistry, St Lukes Campus, Heavitree Road, Exeter EX1 2LU, UK; 3 Imperial College, St Mary's Campus, London, UK

## Abstract

**Background:**

Although young people's transition from Child and Adolescent Mental Health Services (CAMHS) to Adult Mental Health Services (AMHS) in England is a significant health issue for service users, commissioners and providers, there is little evidence available to guide service development. The TRACK study aims to identify factors which facilitate or impede effective transition from CAHMS to AMHS. This paper presents findings from a survey of transition protocols in Greater London.

**Methods:**

A questionnaire survey (Jan-April 2005) of Greater London CAMHS to identify transition protocols and collect data on team size, structure, transition protocols, population served and referral rates to AMHS. Identified transition protocols were subjected to content analysis.

**Results:**

Forty two of the 65 teams contacted (65%) responded to the survey. Teams varied in type (generic/targeted/in-patient), catchment area (locality-based, wider or national) and transition boundaries with AMHS. Estimated annual average number of cases considered suitable for transfer to AMHS, per CAMHS team (mean 12.3, range 0–70, SD 14.5, n = 37) was greater than the annual average number of cases actually accepted by AMHS (mean 8.3, range 0–50, SD 9.5, n = 33).

In April 2005, there were 13 active and 2 draft protocols in Greater London. Protocols were largely similar in stated aims and policies, but differed in key procedural details, such as joint working between CAHMS and AMHS and whether protocols were shared at Trust or locality level. While the centrality of service users' involvement in the transition process was identified, no protocol specified how users should be prepared for transition. A major omission from protocols was procedures to ensure continuity of care for patients not accepted by AMHS.

**Conclusion:**

At least 13 transition protocols were in operation in Greater London in April 2005. Not all protocols meet all requirements set by government policy. Variation in protocol-sharing organisational units and transition process suggest that practice may vary. There is discontinuity of care provision for some patients who 'graduate' from CAMHS services but are not accepted by adult services.

## Background

Even though adolescence is a risk period for the emergence of serious mental disorders such as schizophrenia, it has generally received only patchy attention from services [1]. In UK only 36% of child and adolescent mental health services (CAMHS) have specific teams for adolescents [2]. Psychopathology often continues between adolescent and adult years [3]. Many young people with mental health problems therefore require long-term engagement with health services and are likely to experience transfer of care (hereby called transition) from CAMHS to adult mental health services (AMHS) The term transition has two distinct meanings: a developmental transition, from a life stage such as adolescence to adulthood; or a situational transition, from one health service provider to another [4]. In this paper transition refers only to situational transition i.e. transition of care from CAMHS to AMHS.

Traditionally CAMHS see young people up to the age of sixteen years or up to school-leaving age [5], although over half now offer services up to the eighteenth birthday [6]. This means that some young people of sixteen and seventeen years of age are not receiving the services they require, as most AMHS tend to have a lower age limit of eighteen years [5]. Potential problems of transition are not due to age boundaries alone; there are fundamental differences between CAMHS and AMHS in their theoretical base, service organisation and professional training, all of which impact on the process of transition. [1,7].

In recognition of the importance of transition process, recent UK Government policies have emphasised the importance of transition between child and adult services [5,8,9]. Tools for facilitating such transition in practice [10] and performance indicators to monitor the process [11] have been introduced to ensure successful implementation of policy into practice. However the latest Government guidance on improving the transition from children's to adult health services [12] specifically excludes CAMHS/AMHS transition. Only 23% of mental health services in the UK have specific arrangements for CAMHS to AMHS transition [13] and there is a widespread view that the process of transition is unsatisfactory for users, carers and professionals.

Problems of transition are not limited to the British context [14] and some Australian services have started implementing innovative youth service models that spans the traditional CAMHS-AMHS divide [15]. Despite the obvious importance of successful transition between CAMHS and AMHS, there are very few studies that have attempted to understand the process, outcome and experiences of transition [16].

The TRACK study is a multi-site, mixed methods study that aims to explore policies, processes, predictors and experiences of transition of care. In this paper we present the findings from the first stage of TRACK: a study of Greater London CAMHS' transition protocols. The specific objectives of this stage were to identify existing transition protocols within CAHMS in Greater London; to conduct a content analysis of these protocols and to determine the annual transition rates from CAHMS to AHMS.

## Methods

### Sample

A contemporary list of Greater London CAMHS that potentially referred to AMHS was not available when the TRACK project was started. An organic process was therefore undertaken to identify existing protocols. Between August-December 2004 several sources of information including the National CAMHS Support Service (hosted by the Department of Health), child psychiatrists and service managers were asked to help the TRACK team identify Greater London CAMHS that potentially referred to AMHS. During data collection, this list was sent along with the study tool and respondents asked to provide information about any other CAMHS not on the list. Any further services thus identified were also recruited into the study.

### TRACK Questionnaire

A literature review of transition from child to adult mental health services was undertaken through searches of Medline, EMBASE, CINAHL, PsychINFO, The Cochrane Library, International Bibliography of Social Sciences (IBSS), National Research Register, the HEA Database, and reports and publications from the Department of Health and charities, such as *Young Minds *and *Rethink*. Based on the review, a semi-structured study tool was developed which comprised of two parts: the first sought information on the structure of the respondent organization, e.g. type of service, catchment area, transition boundaries, interface with other services etc. The second part collected information about local transition protocols and estimates of the average annual numbers of young people who were considered suitable for transfer to AMHS, actually accepted by AMHS and remained with CAMHS beyond the transition boundary. A copy of any transition protocol was requested.

For the purpose of this study, a service was defined as a "*provider agency that provides CAMHS tier 2/3/4 services with shared transition protocols and procedures*". The questionnaire specified that "If within your service, some teams use different protocols or procedures for transition, please count each group of teams using a shared transition procedure/policy/protocol as a distinct service".

### Data collection

Lead clinicians and service managers of identified CAMHS were posted a letter explaining the purpose of the study and asked to complete the questionnaire in consultation with the multidisciplinary team. Two further reminder postal requests, supplemented by follow-up telephone calls, were sent to improve recruitment rates.

### Analysis

Data were entered into SPSS and descriptive statistics were produced. Protocols were subjected to content analysis. Key transition-related themes had initially been identified from a specific policy document [17], literature search, sample transition protocols obtained from Trusts outside London, and TRACK study participants. Themes identified (e.g. transition boundary) were allocated to pertinent procedural concepts (e.g. transition criteria and service boundaries). Counts of protocols containing specific themes were thereby generated per procedural concept.

## Results

By April 2005, we had identified 65 CAMHS in Greater London, from which we received 42 (64.6%) completed questionnaires. Responses identified 15 protocols of which 2 were draft versions.

Respondents (n = 42) were located in 11 health Trusts, with each having at least 5 teams (range 5–41, mean 15.7) per CAMHS. Of the non-responding Trusts, 78% CAMHS comprised of only one team. Respondents therefore came from most of the larger CAMHS. Respondents described themselves as 'CAMHS' (20), adolescent mental health services (12), specialist CAMHS (1), specialist adolescent mental health services (2), in-patient CAMHS (1), inpatient adolescent mental health service (1), national CAMHS (4) and national in-patient CAMHS (1), serving populations ranging from 60,000 to 4 million, having 1–37.5 whole-time equivalent staff (mean 10.9, SD 9.02, n = 41) and having between 10 and 1500 currently open cases (mean 438.32, SD 469.56, n = 31).

### Structure of protocol-sharing units

We received 15 protocols of which two (protocols 5 and 12) were draft versions. The protocol-sharing units varied greatly. Protocol 6 was shared by 2 Trusts providing CAMHS, including generic, targeted and inpatient 4 teams. Protocols 1,2,7,8,9,10 and 15 each covered teams within one Trust. In relation to these protocols, responding teams within each protocol-sharing unit varied between being generic, locality teams (protocols 1, 9 and 15); generic teams at locality and wider than locality level (protocol 2); locality-based, adolescent teams targeting specific conditions (protocol 8); a generic team providing for 14–30 year olds at wider than locality level (protocol 7); and generic and targeted locality teams alongside national targeted and tier 4 teams (protocol 10). Within another Trust each of the four generic teams covering different localities had a protocol of their own (protocols 11,12,13,14). Within another Trust three generic locality teams covering the same locality shared one protocol (3); an in-patient unit covering this locality and other areas used two protocols (3 and 5); and a specilaist adolescent team covering used another protocol (protocol 4).

### Transition boundary

The transition boundary between CAMHS and AMHS varied, with 18 years being the modal boundary (n = 25). Among the other protocols, the transition boundary varied as follows: 16-years (n = 2); 17-years (n = 1); 16-years if not in full-time education (NIFTE) or else 18-years (n = 5); 17 if NIFTE or else 18-years (n = 2); 18-years, but up to 19 for young people with certain diagnoses (n = 1); 19-years (n = 2); 20-years (n = 1); and over 21-years (n = 1). One responding team was for children and not for young people and therefore did not interface with AMHS.

The responding teams' estimates of their average annual number of cases considered suitable for transfer to AMHS ranged between 0 and 70 (mean 12.3, SD 14.5, n = 37). Estimates of their average annual number of cases that actually made the transition ranged from 0 and 50 (mean 8.3, SD 9.5, n = 33). Average numbers of service users who continued to be seen by the team beyond the transitional boundary varied from 0 and 64 (mean 7.6, SD 11.8, n = 31).

### Transition protocols

Only the 13 agreed protocols were subjected to content analysis; draft protocols were excluded since we wanted to capture information about ongoing practice. There were several broad similarities between the stated principles of the protocols. Most referred to the National Service Framework documents [5,17,18] and identified the following factors as important in ensuring smooth transition between services: consistency in service, continuity of care, a seamless transition, clarity about professional's roles and clinical responsibility, information sharing between agencies, aligning of assessment processes between services, resolution of eligibility and funding criteria, joint working preceding final transfer, co-operation & flexibility, user and carer involvement in decision making, care based on the principle of informed consent and consideration of the most appropriate care provision for a young person. All protocols considered an enduring mental health problem or the likelihood of mental health needs continuing in to adulthood as important criteria for referral to AMHS. There was therefore very little variation in the stated principles underpinning the protocols.

Table 1 summarises the key differences between protocols. Protocols differed in terms of which services/agencies had been involved in developing the protocols; the transition boundary age and whether this was flexible; the procedure for patients not accepted by AMHS; what information should be transferred; and whether the individual's care level according to the Care Programme Approach (CPA) [19] was a transition criterion. Protocols also differed in relation to specifications for the process of transition such as the duration of any transition-planning period and whether a formal transition plan was to be drawn up. Differences in terms of joint working included whether protocols specified a planning meeting between CAMHS and AMHS to help assess need for transition and agree a transition or discharge plan; the involvement of other agencies in this process and CAMHS input post-transition. Although most protocols (n = 11, 85%) considered discussion with the service user as central to the transition process, none specified ways of preparing the service user for transition.

**Table 1:** Identified differences between transition protocols across Greater London

**Protocol theme n = 13**	n (%)	Further details n (%)
Agencies involved in developing protocol	not specified: 8 (62%)	specified: 5 (38%), from 2 (CAHMS and adult services) to 6 agencies (CAHMS, AMHS, PCT, Social Services, Information technology and Voluntary sector)
CPA used as transition criterion	No: 10 (77%)	Yes: 3 (23%): patients on Enhanced CPA considered appropriate; those on Standard CPA would "be considered"
Transition boundary: 18^th ^birthday	Yes: 9 (69%)	No: 4 (31%): 3 (23%): 16^th ^(n = 2) or 17^th ^(n = 1) birthday if patient not in full time education (FTE), and 18th birthday if in FTE; 1 (8%): transition boundary 21^st ^birthday
Transition boundary flexible	Yes: 10 (77%)	No: 3 (23%)
Specified duration of transition planning	No: 1 (8%)	Yes: 12 (92%): 6 (46%) at least 6 months; 2 (15%) at least 3 months; 4 (31%) at CAMHS review prior to transition
Joint planning meeting	at least one: 11 (85%)	Joint work mentioned in 2 (15%), no details specified
Formal transition plan to be drawn up	Not specified: 5 (38%)	Specified: 8 (62%): 5 (38%) before first appointment with AMHS; 2 (15%) following assessment by AMHS; 1(8%) basic plan before and final plan after assessment by AMHS
Multi-agency involvement in transition planning	Not specified: 5 (38%)	Yes: 8 (62%): 6 (46%) a general remark; 2 (15%) specified inclusion in decision-making and information sharing
Joint working during transition	Not specified: 9 (69%)	Yes: 4 (31%)
Information to be transferred	Risk assessment and management plan: 6 (46%)	Other: 1 (8%) all case notes; 1 (8%) specifically not individual session notes, except where directly relevant e.g. because of high risk levels; 1 (8%) nothing specified; 2 (15%): "significant" reports, e.g. Occupational/Speech anguage Therapy, Psychology; 3 (23%): details of interventions & multi-agency working; 2 (15%): Framework for the assessment of children in need and their families [25]
Procedures for patients not accepted by AMHS	Nothing mentioned: 10 (77%)	2(15%) joint discussion between CAHMS and AMHS on further management; 1 (8%) find 'alternate' AMHS

Two protocols specifically mentioned a transition liaison worker, one between CAMHS/AMHS and one between adolescent and adult in-patient units. Single protocols (8%) mentioned the local availability of a consultation-liaison service, through which CAHMS could request assessments and advice regarding ongoing care without the need for transition; and the need to conduct an assessment of the carers' needs.

## Discussion

### Main findings

By April 2005 there were at least 13 active transition protocols in Greater London. Protocol-sharing units varied between a single Trust and between two Trusts to one or several teams within a locality CAMHS service. In the latter category, units varied between being generic, targeted and inpatient teams. This confirms that organisational variation is not a barrier to establishing transition protocols, although surprisingly some services within the same organisation had more than one protocol. What this study was not designed to answer is whether the variation in protocol-sharing units leaves gaps, i.e. CAMHS/AMHS interfaces that are not covered by agreed protocols, or whether the variation is a result of trying to cover the gaps.

Content analysis of protocols revealed little variation in their underpinning principles, which were based on the National Service Frameworks [18,5,17]. Although most protocols identified the service user as central to the transition process, none specified ways of preparing him/her for transition. This suggests that protocols may have been written more with policy than clinical practice in mind.

Protocols differed on practical aspects of transition, ranging from who was involved with their development to transition boundaries and the process of transition planning, including variations in expected joint working. Three quarters of the protocols had no provision for ensuring continuity of care for cases not accepted by AMHS. The discrepancy in numbers thought suitable for transition and the numbers that actually make the transition raises questions about the outcomes of those who 'graduate' from CAMHS but are not accepted by AMHS. Since only a small proportion of these cases continue to receive care from CAMHS beyond transition boundaries, the outcome of the rest should be a cause for concern for service providers.

All protocols considered an "enduring mental health problem" as an important criterion for referral to AMHS. The term 'enduring mental health problem' seems to be a hybrid of the term 'severe and enduring mental illness', used by adult services, and 'mental health problems', a term used more in CAMHS. Stakeholders in the transition process may well hold differing conceptions of mental health, mental illness or disorder/problems [20-22]. Young people with mental health problems as understood in a developmental or CAMHS context may not fulfil the disorder/illness criteria used by AMHS for prioritising and targeting mental health care. So while individuals with psychosis or severe mood disorder may have their care suitably transferred, others with conduct disorder, ADHD, borderline learning disability, autistic spectrum disorder etc may fall through the care net if not considered suitable for AMHS.

When should the mental health problems of a young person looked after by CAMHS become the responsibility of AMHS? Our data suggests that there is no consensus on this issue with current boundaries based on historical service development reasons rather than evidence or best practice. The variation in boundary definition depending upon educational or employment status is difficult to justify. If adult services are appropriate for unemployed 16 year olds who are still living with their parents, why are adult services not appropriate for 17 year olds who are about to leave the sixth form for university? Mental health services for 16 and 17 year olds are disproportionately expensive – so that comprehensive mental health services for individuals up to their 18th birthday may cost around twice as much as similar services that end at people's 16th birthday [23]. If cost is the reason behind a service gap for 16–18 year olds, then the only way to bridge this gap is to resource services adequately.

Perhaps the best way forward is to develop specialist youth mental health services. McGorry has argued for such services, stating that "public mental health services have followed a paediatric-adult split in service delivery, mirroring general and acute health care. The pattern of peak onset and the burden of mental disorders in young people means that the maximum weakness and discontinuity in the system occurs just when it should be at its strongest" [15]. Our findings suggest that the complexity of service structures, arbitrary service boundaries, variation in protocols and possible policy-practice gap all contribute to such a discontinuity of mental health care for a significant number of young people who experience no or poor transition of care across services. The early psychosis approach, with its span across the CAMHS-AMHS divide and focus on diagnosis and need rather than age cut-offs, is better placed to avoid such discontinuity than traditional service structures.

### Main limitations

At the time of our data collection, there were 11 mental health trusts in Greater London and we received at least one protocol from the catchment area of each mental health Trust. A comprehensive map of CAMHS services was however unavailable. We identified services using information from several sources. Our aim was not to map CAMHS provision but to identify existing protocols. Responding teams in our study varied from generic to targeted and inpatient teams and from locality-based to wider and national teams. While our study may not have captured responses from every relevant CAMHS and hence some selection bias is inevitable, the wide variation in responding teams suggests that the findings are representative of transition issues facing CAMHS in Greater London. Greater London is primarily urban and changes in service delivery are also frequently initiated in the capital. Both these factors may also limit the generalisability of our findings to other parts of the country. Later stages of TRACK will utilise the appropriate CAMHS Mapping Atlases [24] and cover a more diverse area including services covering rural, semi-rural and non-London urban

The existence of a protocol does not necessarily ensure that actual practice adheres to the stated policy. The next stages of TRACK will identify organisational and clinical determinants of effective and poor transition by evaluating the process of transition using a case note survey and a parallel organisational diagnostic analysis within and between health and social care agencies. Detailed case studies will then explore service users', carers' and mental health professionals' views on transition. Comparison of these findings should help answer some of the questions raised by this paper: What are the reasons for discrepancies between potential and actual transitions? Who stays in CAMHS beyond the transition boundary and why? Do variations in protocol-sharing units leave gaps, i.e. CAMHS/AMHS interfaces that are not covered by agreed protocols, or are they a result of trying to cover gaps? Can CAMHS and AMHS develop shared, common criteria for identifying those who need careful transition planning and a successful transfer of care?

## Conclusion

There are several important clinical, organisational and policy-related reasons to ensure that adolescents with ongoing mental health needs experience a successful transition of care into adult mental health services. Government policy in England and Wales explicitly requires services to develop and implement transition policies between child and adult mental health services. Our study shows that by April 2005, there were 13 transition protocols in operation in Greater London. Not all protocols meet all requirements set by the national policy. Variation in protocol-sharing organisational units and transition process suggest that practice may vary. There appears to be discontinuity of care provision for some patients who 'graduate' from CAMHS services but are not accepted by adult services. The health and social care needs and outcomes of this group, who slip through the care net, must become an area of urgent priority.

## Competing interests

The authors declare that they have no competing interests.

## Authors' contributions

SPS, TF, TK and TW participated in the conception and design of the study. SPS also coordinated data collection. All authors participated in the analysis and interpretation of data, drafting and revision of the paper and read and approved the final manuscript.

## Pre-publication history

The pre-publication history for this paper can be accessed here:


